# Bioactivating Silicon (100) Surfaces with Novel UV Grafting of Cyclopropylamine for Promotion of Cell Adhesion

**DOI:** 10.3390/ma11050713

**Published:** 2018-05-02

**Authors:** Jing Yuan Ching, Chieh-Hua Lee, Yit Lung Khung

**Affiliations:** Department of Biological Science and Technology, China Medical University, No.91 Hsueh-Shih Road, Taichung 404, Taiwan; u106078702@cmu.edu.tw (J.Y.C.); u105010409@cmu.edu.tw (C.-H.L.)

**Keywords:** silicon (100) hydride, UV photoionization, surface bioactivation, cell viability assay

## Abstract

In this report, utraviolent (UV) photoionization of cyclopropylamine on silicon (100) hydride was employed to examine interfacing with three different epithelial cell types (MDA-MB 231, AGS and HEC1A). The cellular viability using this novel methodology had been quantified to evaluate the bioactivating potential of this ring-opening chemistry when compared to standardized controls (aminopropyltriethoxylamine, collagen and poly-L lysine). X-ray photospectroscopy (XPS) and atomic force microscopy (AFM) were used to characterize surface chemistry composition, while cell viability and confocal microscopy after 24 h of incubation were performed. Based on the results acquired from this novel ring-opening metastasis process, the promotion of cell adhesion and viability was found to be higher using this chemistry when compared to other conventional control groups, even for the collagen coating, without any observable issues of cytotoxicity.

## 1. Introduction

The parameters for promoting good cell–surface interactions are highly complicated, but in the majority of academic studies so far, most research groups typically passivate and graft bioactive thin films in order to promote good cellular attachment [1,2,3]. Hence, the deliberate tailoring of the surface chemistry is usually considered as an essential step for bioactivating surfaces, especially for bioinert substrates, such as silicon, which is highly useful for its well-understood chemistry as well as its diverse applications. Collagen has often been reported to be an excellent mediator to bioactive surfaces [4,5,6], although there may be certain concerns pertaining to undesirable immunogenic responses using such a biopolymer [7]. These reasons may have provided the impetus towards achieving bioactivation of surfaces via miscellaneous chemistry modifications, especially on bioinert silicon substrates where initial chemical-based surface modifications have always been deemed necessary before biological cells interfacing [4,8]. Typically, passivation of silicon or silica surfaces is commonly performed via silanization, due to the simplicity of the procedure, as well as its inexpensive starting materials [9]. It is common to find amino-based silanes, such as aminopropyltriethylsilane (APTES) being reported for bioactivating silicon surfaces to improve cell-substratum adhesion [10,11]. However, there are still certain drawbacks regarding this surface modification strategy. Firstly, silanes on silicon surfaces tend to cross-link and cannot be described as a monolayer in most cases [12]. Furthermore, it is sometimes inevitable that harsh organic solvents, such as toluene and tetrahydrofuran, are used during the silanization process and these cannot be considered as environmentally friendly [9]. As such, the use of milder ethanol was also often proposed as an alternative, although the susceptibility to self-condensation of the silanes could render the starting point of this silanization reaction unstable [13]. Nonetheless, many researches have persisted on using APTES to bioactivate silicon surfaces principally due to the lack of a viable chemical alternative, as well as the ease in modification process. 

Recently, our group decided to revisit certain fundamental aspects of thermal hydrosilylation of silicon [14,15,16] that would result in the more stable Si-C linkage formed on the silicon surface. Furthermore, we had also proposed a novel grafting method involving the ring-opening metastasis of cyclopropylamine via UV photoirradiation [17]. We further examined the formation of the surface radicals present in UV photoirradiation and we also noticed the absence of surface radicals generated from high temperature, perceived to be contrary to the traditional perception of thermal hydrosilylation. In fact, this issue of thermal hydrosilylation not producing surface radicals has been widely covered by Colletti et al. [18], as well as several of our previous papers [19,20,21,22,23]. Previously, we utilized cyclopropylamine with a highly sensitive ring structure that was susceptible to radicals and, interestingly, we noticed that under UV photoionization, there was evidence for ring-opening metastasis that was recovered in the form of an amino-rich polymer layer [17]. We also further described the possibility of using this amino-rich polymeric layer to promote cell adhesion, and the cell culture data based on this strategy strongly suggested that this approach would be much more efficient than conventional silanization. This novel chemical approach was deemed more suitable as it was undertaken without the use of harsh organic solvents. Hence, to further justify and elaborate on the potential applicability of this novel grafting chemistry, in this report, we examine the interfacing of a series of different cell types (MDA-MB 231, AGS and HEC1A) growing on a silicon substrate that had been passivated via this method. 

**Figure 1:**
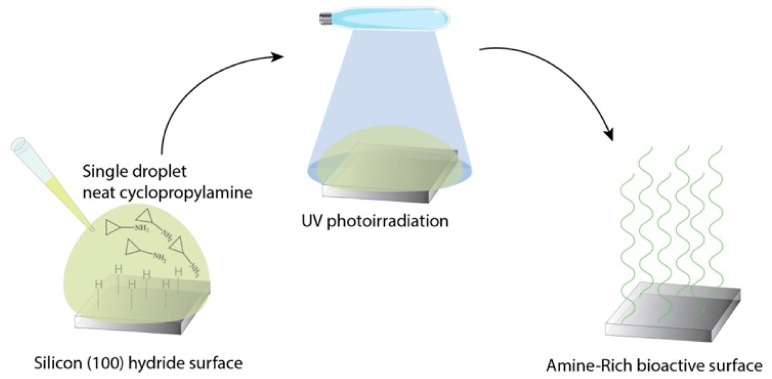
Graphic illustration of the UV photoionization process of cyclopropylamine to generate amine rich bioactive surfaces for cell culture.

In brief, a bare silicon substrate was firstly hydride terminated with a low concentration of hydrofluoric acid, followed by an immediate exposure to UV photoionization with neat cyclopropylamine deposited on the silicon (100) hydrided surface, as shown in Figure 1. In order to expand on the findings from the previous report, we propose here the direct cell-surface application of this rapid UV photoionization for a variety of cell types, as well as a comparison of their cellular viability with other commonly used techniques for producing bioactive surfaces, namely collagen coating, poly-L-lysine grafting and silanization with APTES. X-ray photospectroscopu (XPS) and Atomic Force Microscopy (AFM) were used to characterize the surface chemistry of the UV photoionization process, while overall cellular morphology was determined from confocal and fluorescence microscopy performed to quantify the cell-adhesion profiles of the various cell types in response to this novel surface chemistry. 

## 2. Methods and Materials

Boron-doped silicon (100) wafers, with resistivity of 0.001–0.005 Ω-cm were used in this experiment and were purchased from Semiconductor Wafer, Inc. (SWI, Hsinchu, Taiwan). Unless otherwise stated, all reagents were purchased from Sigma-Aldrich (St. Louis, MO, USA) and were used as received without further purification.

### 2.1. UV Initiated Reaction

The silicon wafers (10 × 10 mm^2^) were prepared by firstly immersing for 30 min into a hot Piranha solution (3 parts 95% Sulfuric acid and 1 part 34.5–36.5% hydrogen peroxide) to clean and rid the surface of physically attached adventitious carbons. The wafer was then dipped into an aqueous 5% hydrofluoric acid for 30 s before being transferred onto a circular quartz disc measuring 1.5 cm in diameter. Subsequently, a volume of 50 µL of cyclopropylamine (neat) was directly added to cover the entire surface. A second quartz disc was placed on top, and the sample was illuminated using a UV source (UVP PenRay model 11SC-1 at an emission of 254 nm at ~2 mW cm^−2^) placed 1 cm above to initiate the reaction for 2 h. 

### 2.2. Silanization Reactions

Unmodified silicon wafers were cleaned with Piranha solution and then rinsed with copious amounts of deionized water prior to silanization. The cleaned silicon wafers were then either immersed in a solution of Aminopropyltriethoxysilane (50 mM in toluene at room temperature) or Hydroxy(polyethyleneoxy)triethoxysilane (PEG, 50 mM in Ethanol at 70 °C) (Gelest, Morrisville, PA, USA). After 2 h, the silicon wafers were sonicated in 95% methanol followed by 95% ethanol and subsequently rinsed with dichloromethane (DCM). The functionalized wafers were then sterilized by immersion in 70% ethanol and exposure to UV light for 30 min.

### 2.3. Collagen and poly-L-Lysine Coating

Cleaning of unmodified pristine silicon wafers was performed by firstly immersing in Piranha solution for 30 min. The silicon wafers were then rinsed thoroughly with deionized water and then blow-dried with argon. After that, 200 μL of 0.1% collagen (Type 1 from Calf Skin, Sigma) was added onto the silicon surface and the reaction was left for 30 min. The coated surface was eventually sterilized by exposure to UV light for 30 min prior to cell seeding. For poly-L-lysine, a neat solution was added to the silicon wafer and incubated for 30 min at room temperature. 

### 2.4. X-Ray Photoelectron Spectroscopy (XPS)

X-ray photoelectron spectroscopy analysis were acquired on a PHI 5000 VersaProbe (ULVAC-PHI) (ULVAC-PHI, Physical Electronics, Chanhassen, MN, USA) equipped with an Al Kα X-ray source (1486.6 eV) and taken at an angle of 45° relative to the substrate. Spectra were also obtained for the C1s, O1s, Si2p and N1s at high resolution scans for all samples. The deconvolution and curve fitting were subsequently performed using XPSpeak while the atomic concentration was determined by CasaXPS (version 2.3.18, http://www.casaxps.com).

### 2.5. Atomic Force Microscopy

Atomic force microscopy (AFM) images were acquired on a Digital Instrument NS4/D3100CL/MultiMode Scanning Probe Microscope (Bruker, Billerica, MA, USA) running on an in-built AFM tapping mode with the cantilever tuned at a frequency of 150 kHz, with a force of 5 N/m in triplicates. The scanned area on the surfaces were 1 μm × 1 μm, and the scan speed was set at 0.6 Hz with the integral and proportional gain set to automatic mode. Image processing was subsequently performed using Gwyddion (MacOS version 2.38, http://gwyddion.net).

### 2.6. Cell Culture

Triple negative breast cancer MDA-MB-231 cells (Cell BRCR number: 60425) and Human gastric adenocarcinoma AGS cells (Cell BRCR number: 60102) were cultured in RPMI, supplemented with 10% fetal bovine serum and 1% penicillin–streptomycin. Uterine endometrium adenocarcinoma HEC1A cells (Cell BRCR number: 60552) were cultured in DMEM F12 supplemented with 10% fetal bovine serum and 1% penicillin–streptomycin. The various silicon samples sized at 1 cm^2^ were placed in a 24-well plate and seeded with an initial density of 3 × 10^4^ cells in static condition for 30 min. Then, the cell medium was removed and the samples were washed with phosphate-buffered saline (PBS) once, followed by re-adding cell medium. These cells seeded in each sample were maintained in a 5% CO_2_ incubator at 37 °C for 24 h before proceeding to the next experiment.

### 2.7. Confocal and Fluorescence Microscopy

To evaluate cell number and morphology, cells on each surface were fixed in 4% paraformaldehyde for 20 min. A 1 μL stock solution of phalloidin-594 (AAT Bioquest, Sunnyvale, CA, USA) was diluted with 100 μL of PBS containing 1% bovine serum as the working solution to stain the cells’ actin filaments. To ensure no excess phalloidin remained, the surfaces were also washed with PBS three times, followed by staining of the nuclei with 1 μL of Hoechst 33342 (AAT Bioquest) in 1 mL of PBS buffer for 10 min. Upon completion of staining, a drop of Fluoromount (NOVUS) was added to the surface, which was then covered with a coverslip and subsequently sealed with nail varnish. The cells with stained actin filaments were observed by confocal microscopy (LEICA SP8) at 40× magnification to examine the cell morphology. To perform the cell count analysis, a total of *n* = 5 surfaces were studied for each condition selected, and five random spots were chosen on each of the surfaces. Fluorescence microscopy images were taken for each of the random spots, and the number of cells was counted. All data were tabulated, and their standard derivations calculated accordingly, in an Excel spreadsheet.

### 2.8. Cell Viability

To verify cell viability on various surfaces, a series of analyses were performed. After 24 h incubation, surfaces with cells seeded were gently moved into a new 24-well plate, and 200 μL of cell medium were added to each well along with 40 μL of assay solution (Cell Meter Colorimetric Cell Cytotoxicity Assay Kit purchased from AAT Bioquest). The solutions were mixed by gently shaking the 24-well plate for 30 s. The surfaces were then incubated at 37 °C in the 5% CO_2_ incubator for 4 h. After the incubation, the absorbance changes in the 24-well plate were observed at 570 and 605 nm in a multiplate reader. The ratio of OD570 to OD605 was used to determine the cell viability in each well. The readings of five replicates were consolidated, and the values were normalized at 100% in conjunction with those from the unmodified control (piranha-cleaned) silicon surfaces. Statistical analysis of cell count differences on the various surfaces was calculated via a one-way ANOVA analysis with relation to the positive control collagen, whereby a *p* value of ≤0.05 was taken as statistically significant.

## 3. Results

**Figure 2:**
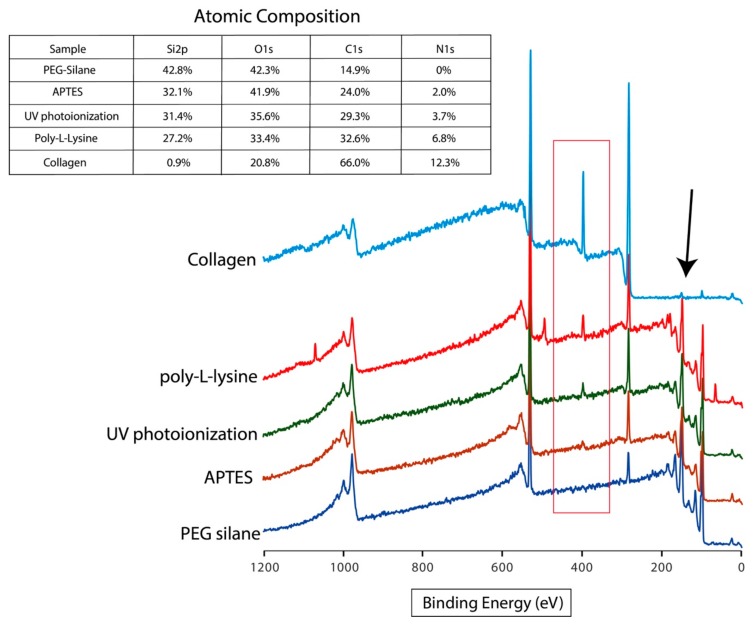
XPS survey spectrum of modified surfaces exhibiting the different N1s concentrations and their respective atomic compositions, as displayed on the inset. Arrows indicate the Si2p region while the box represents the N1s level.

For the comparative study to appreciate this novel photoionization chemistry, we decided to examine cell adhesion and viability on various negative (hydroxyl-terminated unmodified silicon and PEG) and positive control surfaces (APTES, poly-L-lysine and collagen coated) in tandem with the new photoionized surface. Prior to the discussion on the effects of bioactivation, all prepared surfaces were characterized via XPS to investigate their chemical compositions as well as the efficacy of the photoionization chemistry of cyclopropylamine. As shown in Figure 2, the survey spectrum of the modified surfaces revealed that UV photoionization of the cyclopropylamine yielded an N1s concentration of 3.7%, while APTES silanization only produced an overall N1s of ~2%. Understandingly, collagen generated the highest level of N1s at 12.3%, while 6.8% was observed for the poly-L-lysine coating. 

From the XPS, it was clearly evident that the ring-opening metastasis of cyclopropylamine driven by UV photoionization, as reported previously, was able to exhibit a higher concentration of nitrogen content compared to APTES, although it was also noticeable that the surface underwent lesser oxidation in comparison. While the higher oxygen content in APTES and PEG was due to the Si-O linkage and the C-O repeats, respectively, the reasons for the lower nitrogen content in APTES could be primarily due to the cross-linking from the silane side chains that could lower the nitrogen content due to having an unequal coverage of NH_2_. As the surface grafting from the UV photoioinization was polymeric in nature, the nitrogen levels were expected to be higher be higher due to the polymer’s tandem repeats. It was in the author’s opinion that further deconvolution of the polymeric N1s or C1s was not considered useful and therefore, only quantification of the atomic compositions were acquired. Furthermore, it is also important to note that all UV grafting did not produce a highly dense layer on the surface from the survey spectrum Si2p content, unlike collagen which totally passivated the surface masking the silicon signature (Figure 2, black arrow), but this was within our expectations for a covalently grafted polymeric thin film. 

**Figure 3:**
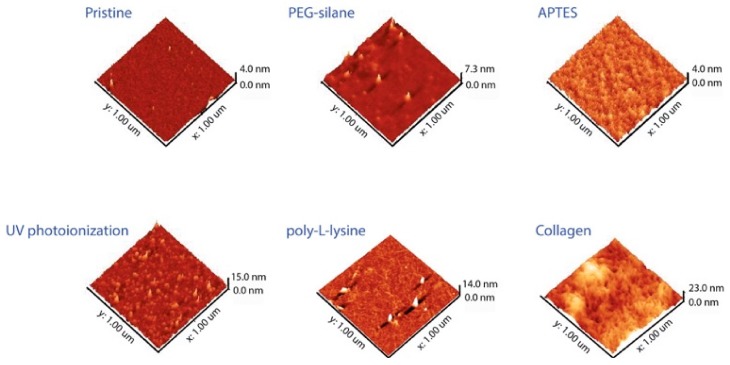
Atomic force microscopy images of all the modified surfaces. Notice that all PEG-silane and APTES modifications did not result in any appreciable roughening on the surface, while UV photoionization exhibits random nano-level patch grafting characteristics.

To further investigate the nature of the generated surface topography, AFM was performed on all surfaces, with the resulting images shown in Figure 3. Both PEG-silane and APTES that underwent the 2 h silanization regime did not produce any considerable modulation on the surface topography compared to unmodified silicon surfaces. On the other hand, randomly grafted nano-level patches were clearly observable for UV photoionization, strongly suggesting a polymeric brush-like feature from chain propagation, similar to our previous report. Poly-L-lysine was found to exhibit a fibrous topography, while thick collagen surface biopolymers were noticeable from AFM analysis. Additionally, UV photoionized surfaces were found to be much rougher (Rms = 1.71 nm) compared to APTES surfaces (Rms = 0.29 nm). This is in part due to the ring-opening polymerization effect that had occurred for the UV photoionized surfaces. Comparisons between both surfaces were deliberately highlighted due to the fact that APTES was often the preferred passivation route in many laboratories for bioactivating surfaces through surface covalent bonding, and that our UV photoionization relied on surface radicals to generate a stable covalent graft. Nonetheless, as the difference in roughness is less than 2 nm (RMS), it is not thought to have had much effect on cell adhesion. Furthermore, in line with the reports by Spatz et al. on the surface placement of amines, as the desensitization of integrins to surface chemical features was found to be ~44 nm [24], enriching the packing density of UV grafted cyclopropylamine would not be as useful to promoting cell adhesion in this study. Although, the authors believed that another means of refining the surface coverage may be to use a highly stringent deoxygenated cyclopropylamine compound to help improve surface grafting and reduce residual oxidation.

**Figure 4:**
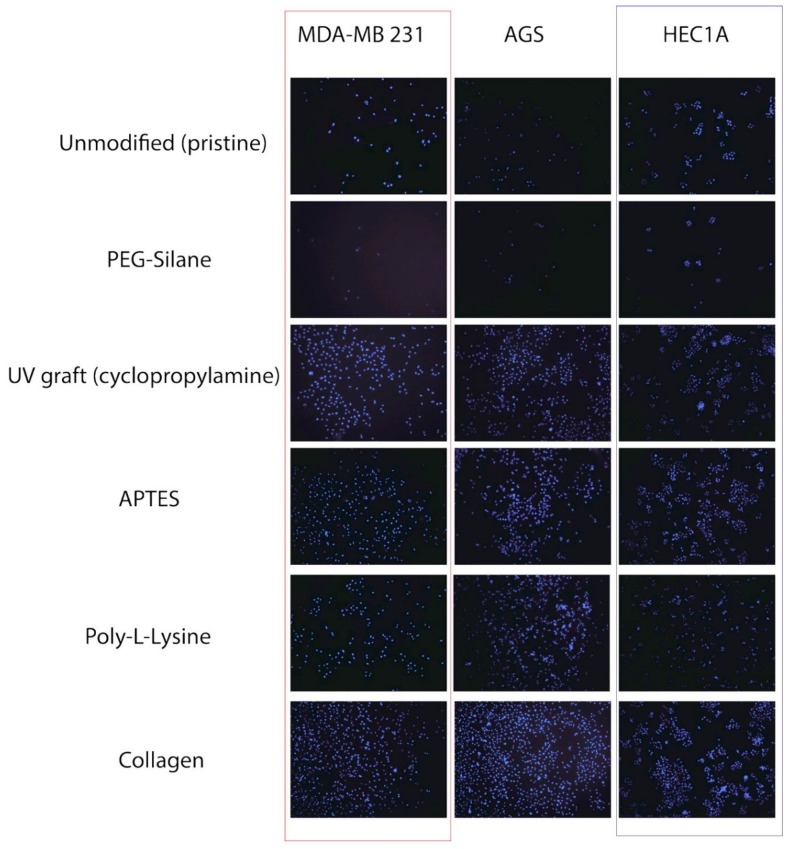
Fluorescence imaging at 10× magnification for all cell types on the various prepared surfaces after 24 h of incubation.

**Figure 5:**
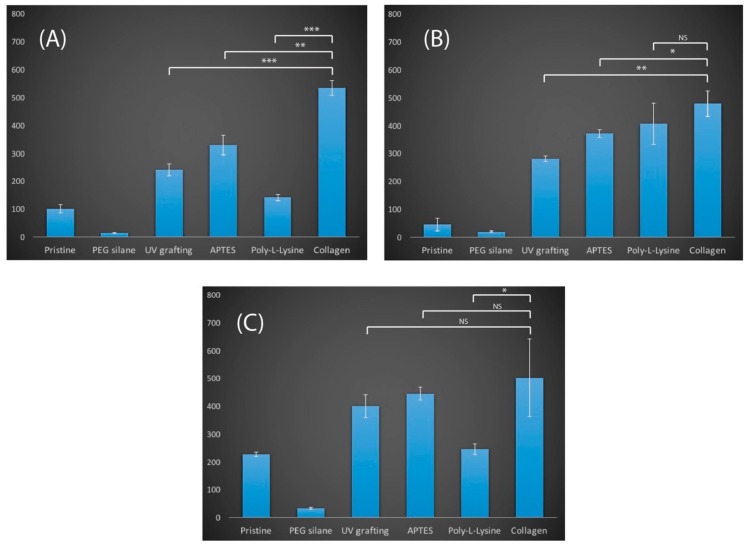
Cell count on the various prepared surfaces after 24 h of incubation for (A) MDA-MB 231, (B) AGS and (C) HEC1A. Statistical significance is denoted as follows: * represents a *p* value ≤ 0.05, ** represents a *p* value ≤ 0.01, and *** represents *p* value ≤ 0.001, while NS represents a value not statistically significant.

In this report, the three epithelial cell lines used as epithelial cell types were very important due to their clinical significance for many therapeutic studies [25,26]. Hence, from an in vitro point of view, there is a need to improve the cellular adhesion to substrates, and this in turn helps to justify their selection in this work. The first visual indicators for cell adhesion were provided from fluorescence imaging as shown in Figure 4. While both PEG and unmodified silicon were within the expectation of having lower cell counts due to their hydrophilic surfaces, UV photoionized silicon surfaces were found to be highly attractive towards adhering cells, similar to APTES and collagen, from both visual observation and cell counts. However, it was clearly noticeable that not all cell types would respond in a similar fashion to the same types of surface modifications. For instance, with poly-L-lysine surfaces, MDA-MB 231 cells (141.13 ± 13.6 cells/mm^2^) were found to have a lower adhesion level compared to AGS (406.22 ± 90.4 cells/mm^2^), while the HEC1A (245.23 ± 23.46 cells/mm^2^) adhesion value was also lower than those of the APTES and the UV grafted surfaces (see Figure 5). In fact, from one-way ANOVA analysis, it seems that the cell adhesion for MDA-MB 231 was highly favorable towards collagen, showing statistical differences to UV cyclopropylamine, APTES and poly-L-lysine. There was little statistical significance for HEC1A cells with respect to the UV cyclopropylamine and APTES surfaces. On the basis of this, such observations strongly suggest that similar surface modifications do not necessarily exhibit similar bioactivating effects to different cell types and this is, in fact, an important finding. This observation was also deemed consistent with the findings reported by Liberio et al. where they observed an increase in bioactivity on poly-L-Lysine coated surfaces, despite their cell types being different [27]. Interestingly, the levels of UV photoionized surfaces remain highly comparative to APTES grafting, although the slight decrease in cell count for all surfaces was attributed to ‘patch-like’ surface grafting, as reported from the AFM (see Figure 3). However, there was no harsh organic solvent used during its preparation, and the reaction was deemed a simpler and cleaner process. The preliminary cell count was also deemed to be highly promising; although, it was necessary to examine the cell viability to check for cytotoxicity of the UV grafting chemistry. 

**Figure 6:**
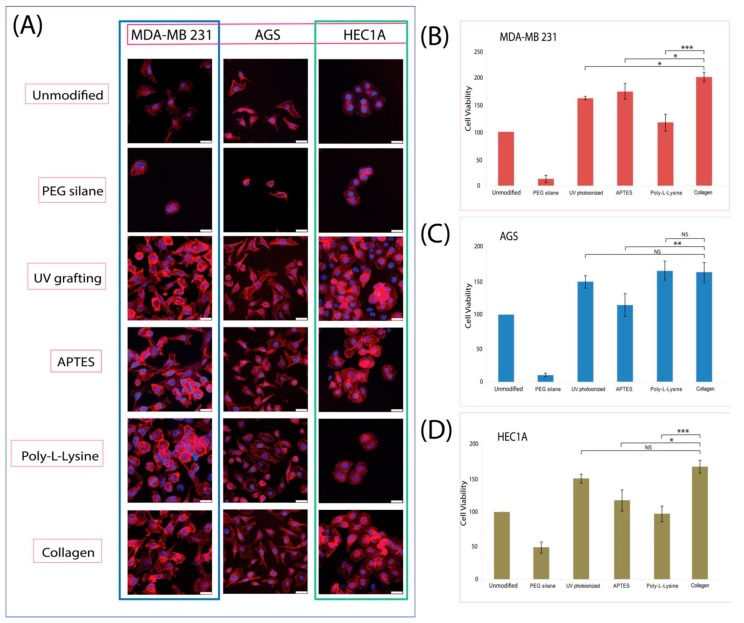
(**A**) Confocal microscopy of the various cells (MDA-MB 231, AGS and HEC1A) on different modified surfaces. Their respective cell viability assay as illustrated on the right for (**B**) MDA-MB 231, (**C**) AGS and (**D**) HEC1A. Statistical significance is denoted as follows: * represents a *p* value ≤ 0.05, ** represents a *p* value ≤ 0.01, and *** represents *p* value ≤ 0.001, while NS represents a value not statistically significant.

As stated previously, in order to examine the cytotoxicity of the various surfaces, the three different cell types were incubated on all surfaces, and the cell viability was determined via a colorimetric based cell viability kit, performed after 24 h of incubation (*n* = 5). At this junction, it is also important to state that we selected these cell types for their differing adhesion morphologies on surfaces in general, with MDA-MB 231 exhibiting good spreading morphology [28] and HEC1A having a rounder overall morphology [29]. Prior to confocal microscopy and cell viability data discussion, it may be necessary to state that the correlation between cell counts from fluorescence and cell viability does not always exhibit a directly proportional relationship. Much depends on the behavior of the adhering cells, although it was noticeable that having such a high cell count for the poly-L-lysine surfaces for the AGS cells correlated strongly with viability (see Figure 6). Even in the case for AGS cells, the levels of cell viability were higher than those for APTES, despite having a much lower cell count. Interestingly, it was evident that for both AGS and HEC1A, there was a higher level of cell viability on UV photoionized surfaces compared to APTES surfaces. For the MDA-MB 231 cells, both sets of data were highly comparable, which was similar to our previous report (Figure 6). Furthermore, for the AGS and HEC1A cells, the performance in terms of cell viability for UV photoionization was highly similar to that of the collagen coating. APTES was not found to have promoted cell viability, which can instead be directly correlated with its high cell count number, and this may be attributed to the harsh chemical regime (toluene) deployed during its synthesis, having a residual effect on the adhering cells. Thus, while APTES may have presented a rich layer of amino on the silicon surface, trace elements of the harsh organic solvents may still be present, adversely affecting the cell viability. On the other hand, the milder UV photoionization grafting of cyclopropylamine was found to have a consistently higher viability regardless of the cell count, and based on this result and the previous chemical analysis from XPS, this may strongly suggest that the polymerization of a thin film of cyclopropylamine-based layer could present a much richer amine decorated surface chemistry that can help in promoting cell adhesion. On the whole, with the exception of MDA-MB 231, UV grafting of cyclopropylamine showed no statistical differences in terms of viability when compared to the positive control collagen, while this was not the case for APTES and poly-L-lysine. Hence, from the cell viability data, we concluded that UV grafting of cyclopropylamine was more universal in terms of promoting cell viability on the surface, and this finding was highly encouraging.

Confocal microscopy analysis of all cell types also revealed some interesting features (Figure 6). Firstly, in terms of surface density, UV grafted cyclopropylamine surfaces were shown to have a much better surface coverage regardless of the cell type used, and the data was highly comparable to that of collagen. It was observed that the cellular morphologies for both UV and collagen surfaces were highly identical. On the other hand, we noticed that the different cells growing on APTES and poly-L-lysine surfaces did not respond in unison, with HEC1A seemingly disfavoring adhesion to poly-L-Lysine as a whole. One interesting observation in our study was that poly-L-lysine was found to have an overall roundish shape for all cell types, and this was also previously observed from our group, although the precise reasons for assuming this shape had not yet been elicited. However, from the morphological perspective, UV grafting had indiscriminately promoted good cell surface interaction regardless of cell type, and this result strongly proposes UV grafting of cyclopropylamine as a highly viable alternative for bioactivating silicon surfaces.

## 4. Discussion

The purpose of this report is to describe a novel and rapid method to produce bioactive surfaces grafted on silicon, and this method is believed to have the potential to replace the more common silanization process. The chemistry had already been previously well covered, and our current XPS data had validated the grafting of the thin film on the silicon surface. It was also important to note that previous degradation studies via highly basic solutions on such UV grafted surfaces had shown a highly resilient surface chemistry as a whole. Hence, it was thought that such surfaces would be highly unsuitable as a replacement for the conventional silanization process. Thus, this report represents the first account of using simple and rapid UV reactions for these biological applications. In order to generate a more holistic profile in terms of bioactivating surfaces, three different epithelial cell types were used, with all of them having consistently presented a highly positive result in terms of cell adhesion number as well as viability. Furthermore, an interesting finding that we have observed from this work is that different cell types respond differently to similar chemistry, and this was most prominent with poly-L-lysine. Considering that L-lysine was a common precursor for bioactivating surfaces, this finding may strongly suggest that caution should be undertaken during the deliberate steps for bioactivating surfaces with coating, as not all cells types would equally respond to a bioactive film. On the other hand, UV photoionization of a thin polymeric graft of cyclopropylamine was seemingly highly bioactive regardless of the cell type, and did not suffer from the residues of harsh chemicals that may have negated its effect. While collagen also shows such ‘universally’ bioactive behavior, due to the abovementioned issues pertaining to immunogenic responses and the instability of a physically absorbed nature, it is not unreasonable to state that collagen grafting especially for use in vivo would not be ideal in most cases. UV grafting of cyclopropylamine, on the other hand, is a covalently grafted thin film that was more stable. Furthermore, our previous examination of its chemistry in harsh basic conditions had strongly suggested that degradation was minimal in most cases, hence rendering this strategy highly viable compared to other chemical modification approaches.

From our results so far, we also suggest that this specific ring-opening metastasis of cyclopropylamine could also be performed as a bioactivating process that can compete well against collagen coating for the promotion of cell adhesion. The main attraction of this work is the inexpensive and environmentally friendly process of producing UV photoionization of cyclopropylamine that can produce a highly bioactive nanolayered film on silicon surfaces, which, in turn, helps in promoting cell adhesion across a wide range of various cell types. This elevates its potential as a viable alternative for the more conventional method of APTES passivation of a silicon substrate.
